# Effect of PERMA Model on Anxiety, Depression, Post-Traumatic Stress Symptoms and Quality of Life in Patients With Scar Plastic Surgery After Burn

**DOI:** 10.62641/aep.v54i3.2182

**Published:** 2026-06-15

**Authors:** Yan Zhong, Zhihui Wu

**Affiliations:** ^1^Department of Plastic Surgery and Burn Center, West China Hospital of Sichuan University, 610041 Chengdu, Sichuan, China

**Keywords:** burns, Cicatrix PERMA Model, psychological distress, quality of life

## Abstract

**Background:**

This study aimed to explore how Positive Emotion, Engagement, Relationships, Meaning, and Accomplishment (PERMA)-based nursing anxiety, depression, post-traumatic stress symptoms (PTSD) and quality of life in patients with post-burn scar plastic surgery.

**Methods:**

A retrospective cohort study was conducted using data from patients who underwent scar plastic surgery for burn-related scar at our hospital between January 2023 and January 2025. Based on the type of nursing care they received, we divided patients into two groups: 51 patients who received PERMA-based nursing were assigned to the PERMA nursing group, and 51 patients who received routine nursing only were assigned to the routine nursing group. Both groups received 12 weeks of nursing care. We assessed all outcome measures, including the Self-Rating Anxiety Scale (SAS), Self-Rating Depression Scale (SDS), PTSD Checklist-Civilian Version (PCL-C), Simplified Coping Style Questionnaire (SCSQ), Self-Acceptance Questionnaire (SAQ), and Generic Quality of Life Inventory-74 (GQOL-74), were assessed at baseline and again after the 12-week nursing period in both groups. Pearson correlation analysis was performed to evaluate the relationships among the psychological improvement scores.

**Results:**

After the nursing intervention, both groups showed decreases in SAS scores, SDS scores, PCL-C scores, and negative coping scores of both groups decreased after the nursing, and those of the PERMA nursing group were lower (*p* < 0.05). The positive coping scores and SAQ scores of both groups increased, and those of the PERMA nursing group were higher (*p* < 0.05). The GQOL-74 scores of both groups increased after the nursing, and those of the PERMA nursing group were higher (*p* < 0.05). Correlation analysis revealed that anxiety relief (ΔSAS) was significantly tied to improvements in both depression and PTSD symptoms. Furthermore, reductions in negative coping strategies (Δnegative coping) acted as a key factor that correlated with lower levels of anxiety and traumatic stress.

**Conclusions:**

PERMA-based nursing is associated with reduced anxiety, depression, and PTSD symptoms, as well as improved coping strategies, self-acceptance, and improved quality of life in patients undergoing scar plastic surgery after burn.

## Introduction

Burns are common and serious traumas, mainly caused by electric current, high temperature, strong radiation, intense radiation or corrosive substances. According to the depth of injury, it can be classified into different severity levels: mild only affects the epidermis, moderate reaches below the dermal papillary layer but leaves some skin appendages, and severe damage involves the entire skin layer and its appendages [1]. Burns not only damage the skin tissue, but also cause pain, infection and dysfunction, and altering the patient’s appearance. These consequences severely impact the quality of life of patients. In particular, the change in body image after scar formation can even trigger intense negative emotions [2]. Burn injuries often lead to hypertrophic scarring and significant psychological sequelae. Research shows that the prevalence of depressive symptoms among hospitalized burn patients ranges from 18% to 45%, with one study finding that 38.8% of these patients experienced depressive symptoms. Burn size was linked to the prevalence of these symptoms, while burn type was not. Patients who had mild depressive symptoms when they were admitted were more likely to recover, while those with severe symptoms showed less improvement over time [3]. Scar repair surgery can improve the appearance of burn patients, but sudden accidents often leave them psychologically unprepared. Patients lack confidence in the surgery, and frequent dressing changes and necessary lifestyle adjustments add further significant psychological stress [4, 5]. Systematic care is a crucial aspect of the rehabilitation process for patients with burn scars and skin deformities.

Routine care for burn patients typically focuses on wound dressing changes, infection prevention and control, and health education . The model focuses on physical rehabilitation but neglects the assessment of psychological needs, which means many patients end up struggling with negative emotions [6]. The Positive Emotion, Engagement, Relationships, Meaning, and Accomplishment (PERMA) model is built around five core dimensions: positive emotions, engagement, interpersonal relationships, meaning, and accomplishment. By systematically fostering these elements, it helps alleviate negative emotions and enhance personal sense of achievement [7]. Previous research has confirmed that the PERMA model can improve the psychological state of parents of premature infants in the neonatal care unit [8]. The PERMA model alleviates the psychological stress of patients with chronic cancer pain [9]. In this study, we aimed to explore how the PERMA model affects anxiety and depression, post-traumatic stress symptoms (PTSD) and quality of life. Our goal is to provide evidence-based nursing plans for patients with burn scar plastic surgery and enhance clinical benefits.

## Methods

### Study Design and Participants

This retrospective cohort study was conducted at West China Hospital of Sichuan University. The study protocol was approved by the Biomedical Ethics Review Committee of West China Hospital of Sichuan University (Approval No.: 2025-2078; Date: November 4, 2025). Written informed consent was obtained from all participants prior to data collection. The study was carried out in accordance with the Declaration of Helsinki.

Inclusion criteria: (1) met the diagnostic criteria for burn scars [10]; (2) had scar contracture requiring plastic surgery and repair; (3) age ≥ 18 years; (4) scar area < 40% of total body surface area, all deep second-degree burns; (5) had complete medical records.

Exclusion criteria: (1) had severe mental disorders (such as schizophrenia, bipolar disorder) that would prevent them from cooperation with assessment; (2) were receiving concurrent systematic psychotherapy; (3) had concurrent hematological diseases; (4) had facial muscle damage from other factors.

Participant selection and grouping: A total of 120 patients who met the eligibility criteria were initially identified from electronic medical records between January 2023 and January 2025. Patients were assigned to two groups based on the type of nursing care they actually received during their hospital stay: PERMA nursing group (n = 51): Patients who received PERMA-based nursing, with data recorded between January 2023 and June 2024. Routine nursing group (n = 51): patients who received routine nursing, with data recorded between July 2024 and January 2025.

### Nursing Measures

Routine nursing group: Patients in this group received standard postoperative care, including oral health education (scar reconstruction techniques, postoperative precautions, prognosis), psychological evaluation with timely guidance for anxiety or depression, answering individual questions, urging timely medication changes, immediately informing the physician of any signs of infection, and advising patients to avoid scratching the affected area. At discharge, patients’ WeChat accounts were added to facilitate consultations after they went home.

PERMA nursing group: In addition to the same routine nursing care, patients also received PERMA-based nursing. The nursing team consisted of five nurses, one psychological counsellor, one head nurse, and one attending physician, each with > 5 years of experience. The head nurse designed the PERMA program, and nurses were responsible for implementing the specific nursing measures.

#### Nursing Timeline (12 Weeks Total)

The average hospital stay was approximately 3 weeks (range: 2–4 weeks). The 12-week nursing period was divided into three phases: Weeks 1–3 (inpatient phase): We delivered stages 1 to 3 of the PERMA programs face-to-face while patients were hospitalized. Each session lasted approximately 30–45 minutes.

Weeks 4–8 (post-discharge practice phase): We started stages 4 to 7 during the later part of patients’ hospital stay. Patients practiced these learned skills independently at home after they were discharged.

Weeks 9–12 (post-discharge follow-up phase): We conducted weekly WeChat follow-ups (voice or video calls) for 4 weeks. Each follow-up session lasted approximately 15–20 minutes and reinforced the content of previous stages.

Importantly, the 4-week WeChat follow-up was embedded within the 12-week nursing period (Weeks 9–12), not an extra period added after the intervention was completed.

#### Stages of PERMA-Based Nursing

The PERMA-based nursing program was delivered in 7 sequential stages.

Stage 1 (Week 1, emotion identification): Patients were interviewed about the impact of burns on their lives and any negative thoughts. Nurses guided patients to express negative emotions and introduced them to dialectical thinking (for example “What good things have come from this situation? What lessons have you learned from it?”).

Stage 2 (Week 2, positive emotion cultivation): Videos and slides explained the concept of positive psychology. Patients were interviewed about people or events they were grateful for after their burn injury. They were instructed to record one positive event every day before bedtime, along with why that event happened.

Stage 3 (Weeks 3–4, positive thinking construction): Examples of positive living after burns were discussed. Interviews focused on nursing gains, positive changes, and future expectations. Patients were guided to imagine their future life and document these in a diary with weekly and monthly plans for themselves.

Stage 4 (Weeks 5–6, engagement and flow experience): Videos and slides explained the concept and significance of flow. Patients were guided to identify personal interests and engage in interest-based activities to achieve a state of flow and relaxation.

Stage 5 (Weeks 7–8, interpersonal relationships): We taught patients effective communication skills. Patients were asked to invite at least one relative or friend for a walk, engage in an in-depth communication, and express their appreciation to that person. We also asked them to either write a thank-you letter or call the person they most wanted to thank.

Stage 6 (Weeks 9–10, meaning of life): Videos and slides explained the concept of life meaning. Interviews explored patients’ sense of meaning and help them assisted in formulating life plans.

Stage 7 (Weeks 11–12, achievement and goal setting): We explained why personal achievements are important for recovery. Successful cases of other patients who had recovered well from burn scars. Achievement-themed interviews were conducted to assist patients in setting specific goals and implementation plans, and we emphasized the importance of family encouragement and support during this process.

#### Quality Control

All team members received standardized training on the PERMA model and the study’s nursing protocols prior to study initiation. To ensure the nursing was delivered correctly, the head nurse filled out a checklist after each session to monitor nursing fidelity. Patient adherence was recorded via attendance logs for the sessions and WeChat response rates.

### Data Sources and Assessment Tools

Retrieve the assessment data of two groups of patients from the hospital’s electronic medical record system.

Anxiety and depression: Before and after the nursing, the Self-Rating Anxiety Scale (SAS) and the Self-Rating Depression Scale (SDS)were used for assessment. Each scale contained 20 items. Each item was scored from 1 to 4, and the total score was calculated by summing up. The standard score was obtained by multiplying the total score by 1.25. The maximum score was 100. The higher the score, the more severe the anxiety and depression [11, 12].

PTSD assessment: The PTSD Checklist-Civilian Version (PCL-C) [13] was used before and after the nursing. The scale with a total of 17 items and each item was scored from 1 to 5, and the total score ranged from 17 to 85. A higher score indicated a more severe degree of PTSD.

Coping styles: The Simple Coping Style Questionnaire (SCSQ) was used before and after the nursing. It contained 8 items for negative coping and 12 items for positive coping. Each item is scored from 0 to 3 points. The higher the score, the stronger the corresponding tendency [14].

Self-acceptance: The Self-Acceptance Questionnaire (SAQ) was used before and after the nursing [15]. It includes the self-evaluation dimension and the self-acceptance dimension, both consisting of 8 items. Each item ranges from 1 to 4 points, with a total score ranging from 16 to 64 points. The higher the score, the greater the degree of self-acceptance.

Quality of life: The Generic Quality of Life Inventory-74 (GQOL-74) was used before and after the nursing to evaluate quality physical function, psychological function, social function, and material living condition [16]. The GQOL-74 consists of four dimensions: physical function, psychological function, social function, and material life state. Each dimension is standardized to a score ranging from 0 to 100, with higher scores indicating better quality of life.

### Statistical Analysis

Given its retrospective design, this study did not conduct an a priori sample size calculation, and all eligible patients screened during the study timeframe were enrolled. The data were analysed using SPSS 27.0 (IBM Corp., Armonk, NY, USA). The measurement data were tested for normality using the Shapiro–Wilk test. Skewed distributions were expressed as M (P25, P75), while normally distributed data were described as $\bar{x}$ ± s. For data that met the normal distribution criteria, paired *t*-tests were used for within-group comparisons, and independent sample *t*-tests were used for between-group comparisons. For the comparison within the non-normal data groups, the Wilcoxon signed-rank test was used; for the comparison between groups, the Mann–Whitney *U* test was employed. The count data were described as percentages and analysed using the χ^2^ test; for the ranked data, the rank sum test was applied. A two-tailed *p* < 0.05 was considered statistically significant. Additionally, Pearson correlation analysis was performed across all participants (N = 102) to evaluate the relationships among improvement scores (Δ, change from baseline to post-intervention) of various psychological dimensions. For symptom scales (SAS, SDS, PCL-C, and negative coping), Δ was calculated as pre-minus-post score, while for capacity scales (positive coping and SAQ), it was post-minus-prescore. Statistical significance was set at *p* < 0.05.

## Results

### Comparison of general data between the two groups

There were no statistically significant differences between the two groups in terms of gender, age, body mass index (BMI), educational level, monthly per capita family income, or burn site (*p* > 0.05) (Table 1).

**Table 1.** **Comparison of two groups of general data**.

Data	Parameters	Routine nursing group	PERMA nursing group	Statistical value	*p*
		(n = 51)	(n = 51)		
Gender	male	27 (52.94)	25 (49.02)	χ^2^ = 0.157	0.592
	female	24 (47.06)	26 (50.98)		
Age (years)		41.35 ± 4.62	40.96 ± 5.10	*t* = 0.405	0.687
BMI (kg/m^2^)		23.22 ± 1.73	23.15 ± 1.65	*t* = 0.209	0.835
Cultural level	junior high school and below	18 (35.29)	15 (29.41)	χ^2^ = 0.642	0.521
	secondary vocational school or high school	20 (39.22)	21 (41.18)		
	college degree or above	13 (25.49)	15 (29.41)		
Monthly per capita	≥ 5000 RMB	20 (39.22)	18 (35.29)	χ^2^ = 0.168	0.682
income of the family	< 5000 RMB	31 (60.78)	33 (64.71)		
Burn site	chest and abdomen	10 (19.61)	9 (17.65)	χ^2^ = 0.185	0.912
	head, face and neck	24 (47.06)	23 (45.10)		
	limbs	17 (33.33)	19 (37.25)		

Note: Data are presented as mean ± SD, n (%). BMI, body mass index; RMB, Renminbi; n, number of patients; PERMA, Positive Emotion, Engagement, Relationships, Meaning, and Accomplishment. χ^2^, chi-square statistic. 1 USD ≈ 7.25 RMB.

### Status of SAS Scores

There was no statistically significant difference in the scores between the two groups before the nursing (*p* > 0.05). After the nursing intervention, the SAS scores intervention, and the reduction in the PERMA nursing group was more significant (*p* < 0.001). As shown in Table 2.

**Table 2.** **Comparison of SAS scores between the two groups (points)**.

Group	Before nursing	After nursing
Routine nursing group (n = 51)	63.35 ± 5.26	51.25 ± 5.33*
PERMA nursing group (n = 51)	63.48 ± 5.17	42.35 ± 4.67*
*t*	0.092	8.501
*p*	0.927	< 0.001

Note: SAS, Self-Rating Anxiety Scale; PERMA, Positive Emotion, Engagement, Relationships, Meaning, and Accomplishment; n, number of patients; *t*, *t*-statistic; *p*, probability value. Compared with baseline values in the same group, *p < 0.05.

### Status of SDS Scores

There was no statistically significant difference in the scores between the two groups before the nursing (*p* > 0.05). After the nursing, the SDS scores decreased in both groups, and the reduction in the PERMA nursing group was more significant (*p* < 0.001). As shown in Table 3.

**Table 3.** **Comparison of SDS scores between the two groups (points)**.

Group	Before nursing	After nursing
Routine nursing group (n = 51)	61.02 ± 5.38	55.50 (48.00, 56.00)*
PERMA nursing group (n = 51)	61.27 ± 4.95	44.00 (41.00, 45.75)*
*t/z*	*t* = 0.102	*z* = 7.121
*p*	0.919	< 0.001

Note: SDS, Self-Rating Depression Scale; PERMA, Positive Emotion, Engagement, Relationships, Meaning, and Accomplishment; n, number of patients; *t*, *t*-statistic; *z*, *z*-statistic from Mann–Whitney *U* test; *p*, probability value. Compared with the baseline values in the same group, *p < 0.05.

### Status of PTSD Scores

There was no statistically significant difference in the scores between the two groups before the nursing (*p* > 0.05). After the nursing, the PCL-C scores of both groups decreased, and the decrease in the PERMA nursing group was more significant (*p* < 0.001). As shown in Table 4.

**Table 4.** **Comparison of PCL-C scores between the two groups (points)**.

Group	Before nursing	After nursing
Routine nursing group (n = 51)	54.33 ± 4.87	37.00 (34.00, 38.00)*
PERMA nursing group (n = 51)	54.62 ± 4.59	25.00 (24.25, 27.00)*
*t/z*	*t* = 0.567	*z* = 8.274
*p*	0.572	< 0.001

Note: PCL-C, PTSD Checklist-Civilian Version; PERMA, Positive Emotion, Engagement, Relationships, Meaning, and Accomplishment; n, number of patients; *t*, *t*-statistic; *z*: *z*-statistic from Mann–Whitney *U* test; *p*, probability value. Compared with the baseline values in the same group, *p < 0.05.

### Status of Negative Coping Scores

There was no statistically significant difference in each score before the nursing (*p* > 0.05). After the nursing, the scores of negative coping in both groups decreased, with the PERMA nursing group having a lower score; the scores of positive coping increased, with the PERMA nursing group having a higher score (*p* < 0.001). As shown in Table 5.

**Table 5.** **Comparison of SCSQ scores between the two groups (points)**.

Group	Negative coping		Positive coping	
	Before nursing	After nursing	Before nursing	After nursing
Routine nursing group (n = 51)	18.50 (17.00, 21.00)	12.00 (11.00, 14.00)*	16.00 (13.00, 17.25)	21.00 (20.00, 23.00)*
PERMA nursing group (n = 51)	19.00 (17.00, 19.75)	7.00 (6.00, 8.00)*	16.00 (14.00, 17.00)	28.00 (26.00, 30.00)*
*z*	0.777	8.001	0.466	7.993
*p*	0.437	< 0.001	0.641	< 0.001

Note: SCSQ, Simplified Coping Style Questionnaire; PERMA, Positive Emotion, Engagement, Relationships, Meaning, and Accomplishment; n, number of patients; *z*, *z*-statistic from Mann–Whitney *U* test; *p*, probability value. Compared with the baseline values in the same group, *p < 0.05.

### Status of SAQ Scores

There was no statistically significant difference in the scores between the groups before the nursing (*p* > 0.05). After the nursing, the SAQ scores of both groups increased, and the increase was more significant in the PERMA nursing group (*p* < 0.001). These results are shown in Table 6.

**Table 6.** **Comparison of SAQ scores between the two groups (points)**.

Group	Before nursing	After nursing
Routine nursing group(n = 51)	31.33 ± 4.12	36.65 ± 4.09*
PERMA nursing group(n = 51)	31.05 ± 4.38	42.38 ± 4.69*
*t*	0.873	6.245
*p*	0.385	< 0.001

Note: SAQ, Self-Acceptance Questionnaire; PERMA, Positive Emotion, Engagement, Relationships, Meaning, and Accomplishment; n, number of patients; *t*, *t*-statistic; *p*, probability value. Compared with the baseline values in the same group, *p < 0.05.

### Status of GQOL-74 Scores

After the nursing, the GQOL-74 scores of both groups increased, and the improvement in the PERMA nursing group was more significant (*p* < 0.001). There was no statistically significant difference in the scores between the groups before the nursing (*p* > 0.05) As shown in Table 7.

**Table 7.** **Comparison of GQOL-74 scores between the two groups (points)**.

Group	Physical function		Psychological function		Social function		State of material life	
	Before	After	Before	After	Before	After	Before	After
	nursing	nursing	nursing	nursing	nursing	nursing	nursing	nursing
Routine nursing	70.35 ± 5.52	86.35 ± 3.87*	55.65 ± 5.24	81.06 ± 5.70*	58.75 ± 6.14	78.43 ± 4.46*	78.73 ± 5.52	87.35 ± 3.60*
group (n = 51)								
PERMA nursing	71.08 ± 6.31	91.24 ± 2.85*	55.38 ± 5.19	88.43 ± 3.91*	58.29 ± 5.93	86.79 ± 4.01*	79.06 ± 6.38	92.13 ± 3.34*
group (n = 51)								
*t*	0.622	7.266	0.261	7.614	0.385	9.954	0.279	6.951
*p*	0.536	< 0.001	0.794	< 0.001	0.701	< 0.001	0.781	< 0.001

Note: GQOL-74, Generic Quality of Life Inventory-74; PERMA, Positive Emotion, Engagement, Relationships, Meaning, and Accomplishment; n, number of patients; *t*, *t*-statistic; *p*, probability value. Compared with the baseline values in the same group, *p < 0.05.

### Correlation Analysis of Psychological Improvements

As illustrated in Table 8, the reduction in anxiety symptoms (ΔSAS) was significantly and positively correlated with both the reduction in depression (ΔSDS) (r = 0.301, *p* = 0.002) and the alleviation of PTSD symptoms (ΔPCL-C) (r = 0.201, *p* = 0.043). Moreover, the decrease in negative coping behaviours (Δnegative coping) exhibited robust positive correlations with the mitigation of anxiety (ΔSAS: r = 0.264, *p* = 0.007) and PTSD symptoms (ΔPCL-C: r = 0.329, *p* < 0.001). In terms of positive psychological constructs, an increase in positive coping (Δpositive coping) was significantly and positively coupled with the enhancement of self-acceptance (ΔSAQ) (r = 0.252, *p* = 0.011). None of the other correlations reached statistical significance (*p* > 0.05).

**Table 8.** **Pearson correlation matrix of psychological improvement scores (N = 102)**.

Variables	ΔSAS	ΔSDS	ΔPCL-C	ΔNegative coping	ΔPositive coping	ΔSAQ
ΔSAS	1.000					
ΔSDS	0.301**	1.000				
ΔPCL-C	0.201*	0.089	1.000			
ΔNegative coping	0.264**	0.098	0.329***	1.000		
ΔPositive coping	0.040	–0.170	–0.119	–0.110	1.000	
ΔSAQ	–0.155	–0.063	–0.110	–0.169	0.252*	1.000

Note: SAS, Self-Rating Anxiety Scale; SAQ, Self-Acceptance Questionnaire; SDS, Self-Rating Depression Scale; PCL-C, PTSD Checklist-Civilian Version. Δ represents the change score (pre-intervention minus post-intervention for SAS, SDS, PCL-C, and negative coping; post-intervention minus pre-intervention for positive coping and SAQ). *p < 0.05, **p < 0.01, ***p < 0.001.

## Discussion

Burn scars cause significant facial disfigurement and impair physical functions as well as mental health. Research has shown that patients have a significantly higher risk of depression than the general healthy population [17]. Scar reconstruction surgery can fix the appearance defects of caused by burns. However, during the nursing process, the dressing changes, social restrictions, and uncertainty of the condition still cause patients to experience anxiety and depression. These psychological problems seriously interfere with work and life, and in severe cases even show tendencies towards suicide [18, 19]. Systematic psychological care is extremely important for patients with burn scar repair. However, routine care mainly focuses on physical rehabilitation and neglects psychological support. This imbalance can easily lead to negative emotions, which directly holds back improvements in their quality of life [20].

The PERMA theory, through structured interviews and positive psychology training, helps patients establish positive disease cognition and enhance their emotional regulation abilities [21]. The post-nursing assessment showed that the SDS and SAS scores of the PERMA nursing group were lower than those of the routine nursing group. The results suggested that PERMA-based nursing was associated with reduced anxiety and depression symptoms of burn scar reconstruction patients, and this finding was consistent with previous study [22]. The PERMA model allows patients to openly their feelings after burn injury, analyse negative thoughts, and gradually correct incorrect beliefs about the disease. It is based on measures such as “recording three good things”, future outlook, and flow experience. This method helps patients develop positive thinking, enjoy a better life, and alleviate anxiety and depression [23, 24]. The multi-dimensional nursing approach of the PERMA model promotes the transition from basic psychological adjustment to the reconstruction of social roles, which effectively alleviating anxiety and depression symptoms [25, 26]. Unlike previous studiehat focused on patients with internal diseases or caregivers, this study targeted burn scar reconstruction patients. This extends the evidence base for the PERMA model’s to the field of burn rehabilitation. However, we need to be cautious when directly comparing our results to those earlier studies, because there are differences in study design, outcome measures, and patient populations between the studies [8, 9, 22].

PTSD symptoms are a typical stress-related mental health issue. They refer to a persistent and delayed psychological pathological state that occurs in individuals after they experience or witnessing death, serious injury, or life-threatening events [27]. Traumatic dressing changes can trigger PTSD symptoms, and chronic postoperative pain can lead to PTSD. In addition, scarring, disfigurement and functional impairment often lead to PTSD symptoms. The research showed that after the nursing, the PCL-C score of the PERMA nursing group was significantly lower than that of the routine nursing group, suggesting that the PERMA model may be associated with reduced PTSD symptoms of burn scar patients. The PERMA model consists of seven nursing stages: emotion recognition regulation, cultivation of positive emotions, cognitive restructuring, restoration flow and attention, strengthening social support, exploring the meaning of life, and managing achievement goals. These stages work together to contribute to the alleviation of PTSD symptoms and promote psychological rehabilitation [28, 29].

After the nursing, the PERMA nursing group had lower negative coping scores than the routine nursing group, while the scores of positive coping scores, SAQ, GQOL-74 were all higher. The therapeutic effects of the PERMA model operate through five psychological pathways: positive emotions broaden attention and build resilience; engagement redirects their focus from trauma and restores their sense of autonomy; interpersonal relationships counter social withdrawal and reinforce self-worth; meaning facilitates post-traumatic growth; and accomplishment enhances self-efficacy through goal achievement. Together, these dimensions synergistically address both emotional and cognitive aspects of distress [30]. Their emotional regulation ability improved, which created the psychological foundation for them to use more effective coping strategies [31, 32]. The PERMA framework also includes a mechanism to help patients experience flow, which guides them to take part in activities they are interested in, and in turn restoring their autonomy. This nursing shifts the focus of attention. It redirects from traumatic thoughts to controllable behaviours. The ability to cope has been enhanced. The persistence of the strategies has been improved. Communication training and gratitude practice both help reduce social withdrawal. The interpersonal module the program specifically addresses concerns about changes in appearance, by helping patients have positive social interactions. Verified social interactions can provide normative feedback. Such interactions help to re-establish their self-awareness and at the same time strengthen one’s self-acceptance [33, 34]. The PERMA model helps to establish positive cognition and interpersonal functions. This effect stems from its systematic approach, which works to improve self-awareness, regulate positive emotions, and build multi-dimensional resources [35, 36]. As a result, patients feel understood and supported, which makes them more willing to actively cooperate with their care. PERMA-based nursing was associated with improvements in anxiety, depression, and PTSD symptoms, as well as enhanced coping strategies, self-acceptance, and quality of life. However, this study still has certain limitations. For instance, the relatively small sample size (51 per group) limits statistical power and may have prevented detection of smaller but clinically meaningful differences. Moreover, the short follow-up period (12 weeks) precludes assessment of long-term maintenance of nursing effects, and the absence of post-discharge follow-up beyond four weeks limits understanding of durability. The retrospective cohort design inherently limits causal inference.

Our additional Pearson correlation analysis provides key insights into how post-burn psychological rehabilitation works as an interconnected process, rather than separate changes in individual symptoms. The significant positive correlations between ΔSAS, ΔSDS, and ΔPCL-C validate that emotional distress and traumatic stress in scar plastic surgery patients are intertwined clinical entities; hence, an improvement in the affective domain cascades into broader trauma relief. Crucially, our results reveal that the reduction of negative coping (Δnegative coping) serves as a major statistical pivot closely linked to the alleviation of anxiety and traumatic stress. Within the PERMA framework, fostering positive emotions and a sense of meaning directly helps patients disengage from avoidant or maladaptive coping patterns. As negative coping decreases, patients’ cognitive load is reduced, which in turn lowers their acute anxiety and PTSD symptoms. Concurrently, the significant link between Δpositive coping and ΔSAQ suggests that building proactive behaviours and rebuilding self-worth are mutually reinforcing mechanisms. These findings suggest that comprehensive nursing protocols should target both the reduction of maladaptive behaviours and the promotion of positive cognitive traits to systematically optimize clinical outcomes.

Although the two groups were similar in terms of basic demographic and clinical characteristics, we did not control for some unmeasured confounding factors in our analysis. These factors include burn severity (depth, extent, location), pain intensity, the type of surgical procedure, length of hospital stay, postoperative complications, pre-existing psychological resilience, and baseline social support—were not controlled for in the analysis and may have influenced the outcomes. The temporal separation between the observation and routine nursing groups may introduce time-related confounding factors. For example, clinical care practices or patient characteristics may have changed gradually over the study period. It is recommended that subsequent studies improve the research design by expanding the sample size and extending the follow-up period and further explore the nursing mechanism of the PERMA model in patients with burn scars, and comprehensive measurement of potential confounders to suggest and extend these findings. This will provide an evidence-based basis for optimizing clinical nursing plans and improving the clinical benefits for these patients.

## Conclusions

This retrospective cohort study found that PERMA-based nursing is associated with lower levels of anxiety, depression, and post-traumatic stress symptoms in burn patients after they undergo scar reconstruction surgery. It is also associated with more positive coping strategies and higher levels of self-acceptance and overall quality of life. These findings suggest the clinical value of adding positive psychological interventions like the PERMA model to the routine care we provide for burn patients. Future research with larger sample sizes, longer follow-up periods, and more rigorous control of confounding variables is needed to further validate the long-term associations and broader applicability of the PERMA model in this population.

## Availability of Data and Materials

All clinical data included in this study can be obtained by contacting the corresponding author if needed.
